# Diethyl Aminoethyl Hexanoate Priming Ameliorates Seed Germination via Involvement in Hormonal Changes, Osmotic Adjustment, and Dehydrins Accumulation in White Clover Under Drought Stress

**DOI:** 10.3389/fpls.2021.709187

**Published:** 2021-07-29

**Authors:** Muhammad Jawad Hassan, Wan Geng, Weihang Zeng, Muhammad Ali Raza, Imran Khan, Muhammad Zafar Iqbal, Yan Peng, Yongqun Zhu, Zhou Li

**Affiliations:** ^1^College of Grassland Science and Technology, Sichuan Agricultural University, Chengdu, China; ^2^College of Agronomy, Sichuan Agricultural University, Chengdu, China; ^3^Soil and Fertilizer Research Institute, Sichuan Academy of Agricultural Sciences, Chengdu, China

**Keywords:** drought, antioxidants, osmotic adjustment, hormonal regulation, dehydrin accumulation, oxidative damage

## Abstract

Drought is a serious outcome of climate change reducing the productivity of forage species under arid and semi-arid conditions worldwide. Diethyl aminoethyl hexanoate (DA-6), a novel plant growth regulator, has proven to be involved in the amelioration of critical physiological functions in many agricultural crops under various abiotic stresses, but the role of the DA-6 in improving seed germination has never been investigated under drought stress. The present study was carried out to elucidate the impact of the DA-6 priming on seeds germination of white clover under drought stress. Results showed that seed priming with the DA-6 significantly mitigated the drought-induced reduction in germination percentage, germination vigor, germination index, seed vigor index, root length, shoot length, and fresh weight after 7 days of seed germination. The DA-6 significantly increased the endogenous indole-3-acetic acid, gibberellin, and cytokinin content with marked reduction in abscisic acid content in seedlings under drought stress. In addition, the DA-6 significantly accelerated starch catabolism by enhancing the activities of hydrolases contributing toward enhanced soluble sugars, proline content and ameliorated the antioxidant defense system to enhance the ability of reactive oxygen species scavenging under drought stress. Furthermore, exogenous DA-6 application significantly increased dehydrins accumulation and upregulated transcript levels of genes encoding dehydrins (*SK2, Y2SK, or DHNb*) during seeds germination under water deficient condition. These findings suggested that the DA-6 mediated seeds germination and drought tolerance associated with changes in endogenous phytohormones resulting in increased starch degradation, osmotic adjustment, antioxidants activity, and dehydrins accumulation during seed germination under water deficient condition.

## Introduction

Climate change in particular global warming results in significant increases in the frequency and severity of heat and drought stress worldwide. Drought has severely disrupted agricultural production and the coming drought will further extend these loses in the future (Farooq et al., 2009; Farooq et al., 2013). Seed germination is one of the most critical phases of life cycle as it provides the foundation for plant formation. However, water deficiency distracts normal physiological and metabolic activities during seeds germination leading to reduced germination rate and seedling growth (Bai et al., 2020). At this stage, carbohydrates, fats, and proteins metabolism supply energy for seeds germination and subsequent seedling growth (Jie and Kentian, 1993). On the other hand, different types of solutes including water soluble carbohydrates (i.e., sucrose, glucose, and fructose), sugar alcohols (mannitol), proteins (dehydrins), and free amino acids (proline) tend to accumulate, which play a vital function in osmotic adjustment (OA) for reducing water potential of cells during seed germination under water deficient condition (Cao et al., 2018). These solutes also perform function as antioxidants for reactive oxygen species (ROS) scavenging, membrane protection, and other defense mechanisms under drought stress (Bartels and Sunkar, 2005; Seki et al., 2007).

Hormonal regulation is one of the most important and necessary factors for seed germination. Phytohormonal metabolism, stability, and interaction such as abscisic acid (ABA), cytokinin (CTK), gibberellin (GA), and indole-3-acetic acid (IAA) are believed to be strongly associated with seeds germination and stress responses (Miransari and Smith, 2014; Llanes et al., 2016; Zhou et al., 2020). ABA is a primary stress hormone that induces seed dormancy and hinders seed germination. Moreover, ABA regulates stomatal closure to restrict transpiration and water loss in young seedlings under drought stress (Finkelstein et al., 2002). Previous studies reported that CTK could improve seed germination, early seedling morphogenesis, antioxidative defense system, and carbohydrates metabolism in plants leading to the enhanced ROS detoxification, photosynthesis, and OA under water limited condition (Nikolić et al., 2006; Merewitz et al., 2011, 2012). GA serves a plant growth regulator and facilitates in breaking seed dormancy, and IAA improves the emerging radicle growth during the process of seed germination (Vanstraelen and Benková, 2012; Shu et al., 2018). A previous study has shown that exogenous polyamines application ameliorated seed germination and growth of young seedling via alteration of endogenous phytohormones in cotton (*Gossypium hirsutum*) under drought stress (Yang et al., 2016). Moreover, seed priming with sodium chloride enhanced germination of white clover (*Trifolium repens*) seeds by regulating hormonal and carbohydrate metabolism under water stress (Cao et al., 2018).

Diethyl aminoethyl hexanoate (DA-6), a synthetic tertiary amine, is a novel plant growth regulator (PGR) that has been used extensively in many agricultural crops including cotton, pakchoi (*Brassica rapa* subsp. *chinensis*), soybean (*Glycine max*) and maize (*Zea mays*) in the last few years (Jiang et al., 2012; Qi et al., 2013; Liu et al., 2019). Previous studies have shown that the DA-6 exerted multiple advantageous effects such as improvement in seed germination, seedling establishment, photosynthetic rate, grain yield and biomass accumulation on various summer crops (Qi et al., 2013; Liu et al., 2019; Zhou et al., 2019). The DA-6 is well known for its progressive role in defense system of plants under various environmental stresses including cold stress and heavy metal toxicity (Fu et al., 2011; He et al., 2014; Li et al., 2018b). DA-6 can effectively mitigate salt stress through alleviation of oxidative damage in medicinal plants (Zhang et al., 2016). Foliar spray of DA-6 plays an important role in enhancing Cd-extraction efficiency and mitigating heavy metal stress (He et al., 2015). DA-6 application can enhance the growth of micro-algae (*Haematococcus pluvialis*) and increase the quality and quantity of lipids for biodiesel production (Ding et al., 2019). Moreover, combined application of DA-6 with ethephon has also shown its promising effects on various physiological processes such as enhanced mechanical strength of stalk vascular bundles and improved grain yield in maize plants (Xu et al., 2017). Although, numerous studies have described beneficial effects of DA-6 in physiological and metabolic mechanisms under unfavorable environmental conditions, the possible ameliorative effect of DA-6 on physiological, metabolic, and molecular mechanisms during seeds germination has not been discussed under drought stress so far.

White clover is an imperative forage legume cultivated worldwide because of its high crude protein content and excellent nitrogen-fixation potential, hence contributing toward animal nutrition and soil fertility. However, due to its shallow tap-root system and inefficient transpiration control, it is severely affected by drought stress (Annicchiarico and Piano, 2004). Therefore, understanding and amelioration of drought tolerance in white clover are indispensable to enhance legume production, forage quality and quantity, and animal performance all over the world. The aims of this current study were (1) to elucidate the impact of seeds priming with DA-6 on germination characteristics and (2) to disclose DA-6-mediated drought tolerance linking with hormone regulation, osmotic adjustment, starch metabolism, antioxidant defense, and dehydrin accumulation during seeds germination under drought stress.

## Materials and Methods

### Plant Materials and Treatments

White clover seeds (cultivar “Ladino”) were utilized. Seeds were sterilized with 75% ethanol and washed with deionized water for 5 min before being sown. For seeds priming, two set of treatments were used for this study. One set of seeds was drenched in deionized water as control for 3 h at 20°C while the other set of seeds was kept in deionized water for 1 h and later drenched in 2 mM DA-6 solution for 2 h at 20°C. After this, seeds priming with or without DA-6 were placed in plastic containers having three layers of filter papers under deionized water or 17% (w/v) PEG 6,000 (—0.3 Mpa) for drought stress. The plastic containers were incubated in a plant growth chamber with 700 μmol m^–2^ s^–1^ photosynthetically active radiation and average temperature of 23/19°C (day/night) for one week. Each treatment [well-watered control (C), Control + DA-6 (C + D), PEG (P), or PEG + DA-6 (P + D)] included 6 containers (replications) with 100 seeds for each container and arranged in completely randomized design. Seedlings were sampled at 3 and 7 days after germination to examine physiological and biochemical parameters, and germination parameters, fresh weight (FW), shoot length (SL), and root length (RL) were measured at 7 days.

### Measurement of Seed Germination Parameters

Germination vigor (GV) or germination percentage (GP) was calculated at 3 and 7 days of germination, respectively. GV = Number of seeds germinated on 3rd day/Total number of seeds initially sown × 100%; GP = Number of seeds germinated on 7th day/Total number of seeds initially sown × 100% (Li et al., 2007; Thabet et al., 2018). Mean germination time (MGT) and germination index (GI) were evaluated using following formula: MGT = (Ti)(Ni)/ (Ni), where (Ni) is the number of newly germinated seeds in time (Ti), and GI = (Gt)/(Tt), where (Gt) is the number of seeds germinated at “t” day after sowing, while (Tt) stands for time corresponding to (Gt) in days (Zhang et al., 2007). After 7 days of germination, seedling RL, SL, FW, and seed vigor index (VI) were determined. VI was estimated by using GV and seedlings FW, respectively (Li et al., 2014).

### Determination of Endogenous Phytohormones

To measure endogenous IAA, GA and ABA, fresh seedlings (0.4 g) were mechanically ground with 1% glacial acetic acid and 3 ml of methanol plus isopropanol solution (*1:4. v/v*). The mixture was kept in a refrigerator at 4°C for 1 h in dark condition, followed by centrifugation at 8,000 g for 15 min at 4°C. The 2 ml of supernatant was collected, dried, and subsequently dissolved in methanol (300 μl). Afterward, the reaction mixture was exposed to a filtration process by passing it through (0.22 μm) poly tetra fluoroethylene filter (Müller and Munné-Bosch, 2011). Endogenous IAA, GA and ABA concentration were noticed by using Waters Acquity UPLCSCIEX Se-lex ION Triple Quad 5500 System mass spectrometer (Waters, Milford, MA, United States). Samples (5 μl) were inserted into a hoop and loaded onto an Acquity UPLC BEH C18 column (1.7 μm, 50 × 2.1 mm; Waters, Wexford, Ireland) at 40°C. The CTK content was measured by using enzyme linked immunosorbent Assay (ELISA) Kit purchased from *Beijing Fang Cheng Biological Technology Co., Ltd., Beijing, China*.

### Measurement of Carbohydrate Metabolism, Proline and, Osmotic Adjustment

For the estimation of water-soluble carbohydrates (WSC), procedure was conducted according to the protocols of Fu and Dernoeden (2009). 0.5 g of seedlings were randomly sampled and dried in an electric oven. 0.05 g of dried tissue samples were kept in a centrifuge tube (10 ml) and then 80% C_2_H_5_OH (6 ml) was supplemented. Afterward, mixture was heated at a high temperature in a water bath (80°C) for 30 min, cooled rapidly and subsequently centrifuged at 12,000 g for 10 min. The supernatant was collected and utilized for the measurement of WSC, while the residue left was used for the analysis of starch content, respectively. The Activities of amylase enzymes were estimated by following the method described by Tárrago and Nicolás (1976) and Kishorekumar et al. (2007). 0.1 g of fresh seedlings were mechanically ground with 2 ml of deionized water at 4°C. The homogenate was centrifuged at 12,000 g for 25 min at 4°C. The obtained supernatant was utilized for determining α- and β-amylase activities. The 1 ml of 3 mM calcium chloride and 1 ml of supernatant were mixed and kept at 70°C for 5 min. The reaction solution containing 0.1 mM citrate buffer, 0.7 ml of hot enzyme extract and 2% soluble starch solution was placed in an electric oven at 30°C for 6 min and later kept at 50°C for 5 min. The α-amylase activity was measured by using a spectrophotometer (540 nm). The β-amylase activity was determined after the inactivation of α-amylase at pH 3.4. The reaction mixture comprising of 0.1 mM citrate buffer (2 ml), EDTA treated enzyme extract (0.7 ml), and 2% soluble starch solution was kept in an electric oven at 30°C for 5 min after the addition of starch. The β-amylase activity was then measured in similar way as illustrated for α-amylase. Free proline content, seedlings (0.1 g) were taken and homogenized in 35% sulphosalicylic acid (10 ml) to make a fine paste (Bates et al., 1973). Afterward, the homogenate was followed by centrifugation for 10 min and supernatant (2 ml) was mixed with acid ninhydrin solution (2 ml) and glacial acetic acid (2 ml). The reaction mixture was kept in a water bath for 1 h and the reaction was ceased by placing the mixture in ice-bath. After this, toluene (C_7_H_8_) was added, and the absorbance value was noted at 520 nm. To measure the osmotic potential (OP), seedlings were randomly sampled and instantly immersed in deionized water for 8 h at 4°C. Later, seedlings were thawed for 25 min at 4°C to obtain the cell sap for the estimation of the osmolarity using an osmometer (Wescor, Logan, UT, United States). The OP was converted based on the following formula: MPa = −c × 2.58 × 10^–3^ (Blum, 1989).

### Estimation of Enzymatic Antioxidants Activities and Oxidative Injury

For enzyme extraction, samples were mechanically ground with 50 μM cold phosphate buffer (4 ml, pH 7.8) comprising of 1% (*w/v*) polyvinylpyrrolidone. Afterward, the homogenate was centrifuged at 12,000 g for 30 min at 4°C. The supernatant was collected and utilized for the analysis of antioxidant enzyme activities, hydrogen peroxide (H_2_O_2_) content, superoxide radical (O_2_.^–^), and malondialdehyde (MDA) content. Superoxide dismutase (SOD) activity was estimated spectrophotometrically by noting the declining rate of p-nitroblue tetrazolium chloride at an absorbance value of 560 nm (Giannopolitis and Ries, 1977). Other antioxidant enzyme activities including ascorbate peroxidase (APX), peroxidase (POD), and catalase (CAT) were also spectrophotometrically estimated by recording the changes in absorbance values at 290, 470, and 240 nm respectively (Chance and Maehly, 1955; Nakano and Asada, 1981). Protein content was measured using the procedure described by Bradford (Bradford, 1976). MDA content was determined following the protocol of Dhindsa et al. (1981) with minor modifications. 0.5 ml of enzyme extract and 1 ml of reaction solution consisting of 0.5% (*w/v*) thiobarbituric acid and 20% (*w/v*) trichloroacetic acid (TCA) were supplemented and mixed. The solution was kept in a boiling water bath at 95°C for 15 min and cooled rapidly in an ice water bath. The homogenate was centrifuged at 8,000 g for 10 min. Supernatant was collected and absorbance value was recorded at 532, 600, and 450 nm. The rate of O_2_^–^ formation was estimated using sulfanilamide (C_6_H_8_N_2_O_2_S) procedure (Elstner, 1976) and the absorbance value was recorded at 530 nm. H_2_O_2_ content was measured by following the potassium iodide method. The product of oxidation was recorded at 390 nm (Velikova et al., 2000).

### Gene Expression Analysis

To detect transcript levels of genes, real-time quantitative polymerase chain reaction *(qRT-PCR)* was used. For total RNA extraction, fresh seedlings (0.1 g) were extracted by using RNeasy Mini Kit *(Qiagen)* according to the manufacturer’s instructions. After this, a revert Aid First Stand cDNA Synthesis Kit *(Fermentas)* was used for the reverse transcription of RNA to cDNA. Total RNA (500 ng) was utilized for each cDNA synthesis. The cDNA was subjected to *qPCR* using primers of antioxidant enzyme genes *(FeSOD, MnSOD, Cu/ZnSOD, POD, CAT*, and *APX*; Li et al., 2016b) and dehydrin genes (*SK2, Y2SK*, and *DHNb*) (Li et al., 2016a; Table 1). The *PCR* conditions for all above mentioned genes were as follows: 5 min at 94°C, denaturation at 95°C for 30 s (40 repeats), annealing at 56–66°C for 30 s, and extension at 72°C for 30 s (Table 1). The transcript level of genes encoding antioxidant enzymes and dehydrins was calculated using the formula 2^ΔΔCt^ illustrated by Livak and Schmittgen (2001).

**TABLE 1:** Primer sequences and their corresponding GeneBank accession numbers of the analyzed genes.

**Target gene**	**Accession No.**	**Forward primer (5′–3′)**	**Reverse primer (5′–3′)**	**Tm (°C)**
*FeSOD*	KP202173	ACACGATTTCTCAGGGTTACGAC	GCGGCCAAGACTATCAGTTCCAT	58
*MnSOD*	JQ321598.1	TAAGGGAACCTACCCGATAACT	CCAGGACCAAACGTCACCAAAG	66
*Cu/ZnSOD*	JQ321597.1	AACTGTGTACCACGAGGACTTC	AGACTAACAGGTGCTAACAACG	58
*POD*	JQ321606.1	CACTTGGTTTAGTTTTGTCGCC	AACACGGTCTTGTCTGCTACG	64
*CAT*	JQ321596.1	AACAGGACGGGAATAGCACG	ACCAGGTTCAGACACGGAGACA	58
*APX*	JQ321599.1	TAAAGATAGTCAACCCACCTCAACA	ACCAGTCTTGGGAAACAACGTA	58
*SK2*	GU443960.1	TGGAACAGGAGTAACAACAGGTGGA	TGCCAGTTGAGAAAGTTGAGGTTGT	58
*Y2SK*	GU443965.1	GTGCGATGGAGATGCTGTTTG	CCTAATCCAACTTCAGGTTCAGC	60
*DHNb*	GU443960.1	TCCAGTCATCCAGCCTGTTG	CCAGCCACAACACTTGTCA	60
β*-Actin*	JF968419	TTACAATGAATTGCGTGTTG	AGAGGACAGCCTGAATGG	58

### Western Blot Analysis

For western blot analysis, fresh seedlings (0.5 g) were extracted in cold 100 mM Tris-HCl buffer *(pH 8.0)* to get the soluble proteins. The obtained soluble proteins were centrifuged at 12,000 g for 10 min at 4°C. The supernatant was collected and heated in a water bath for 10 min. The supernatant was centrifuged again at 12,000 g and the sediment (equivalent to 30 μg proteins) was utilized for the estimation of dehydrins. To transfer the SDS-PAGE *(12%)* to PVDF membranes, *the Bio-Rad mini protean transblotter* was used. After 2 h of transfer at 65 V and 4°C, the membranes were congested in TRIS-buffered saline for 1 h (Khedr et al., 2003; Vaseva et al., 2011). Later, the TRIS-buffered saline was withdrawn and the PVDF membranes were rinsed shortly in TTBS for 3 times each (5 min). The rinsed PVDF membranes were incubated in *rabbit anti-dehydrins dilution (1:1,000)* for 1 h. Afterward, the membranes were washed in TTBS again for 3 times (5 min) and incubated in *goat anti-rabbit lgG antibody (1: 2,000)* for 1 h. After rinsing in TTBS (20 min), the dehydrins bands were observed by using *TMB* reagent kit *(Sigma, Kawasaki, Japan)* (Close et al., 1993).

### Statistical Analysis

All data was evaluated using statistix 8.1 (version, 8.1. Statistix, Tallahassee, FL, United States). Significant differences among different treatments were estimated with one-way ANOVA in combination with LSD test at the 5% probability level (*p* < 0.05).

## Results

### Growth and Germination Parameters

**FIGURE 1:**
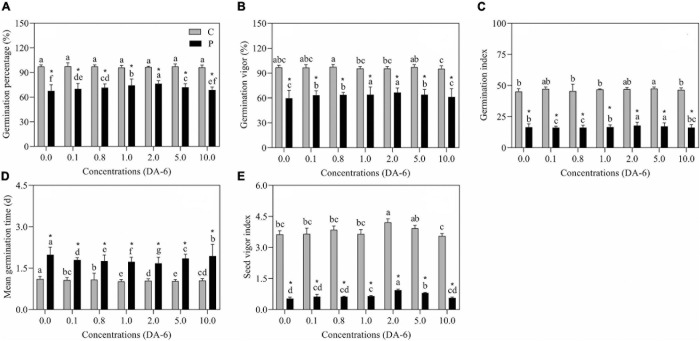
Effect of seed priming with different concentrations of DA-6 on **(A)** germination percentage (GP), **(B)** germination vigor (GV), **(C)** germination index (GI), **(D)** mean germination time (MGT) and **(E)** seed vigor index (SVI) during 7 days of germination in white clover under well-watered condition and drought stress. Different letters represent significant differences among treatments with the application of different concentrations of DA-6 under well-watered condition or drought stress (*p* ≤ 0.05). Vertical bars show the ±SE of mean (*n* = 6), whereas *indicates significant difference between well-watered condition and drought stress under one particular concentration of DA-6.

**FIGURE 2:**
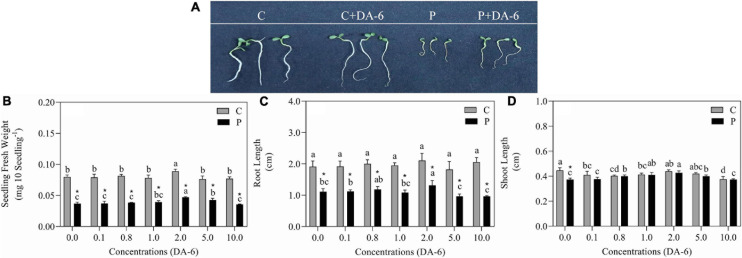
Effect of seed priming with different concentrations of DA-6 on **(A)** phenotypic change, **(B)** seedling fresh weight (FW), **(C)** root length (RL), and **(D)** shoot length (SL) after 7 days of germination in white clover under well-watered condition and drought stress. Different letters represent significant differences among treatments with the application of different concentrations of DA-6 under well- watered condition or drought stress (*p* ≤ 0.05). Vertical bars show the ± SE of mean (*n* = 4), whereas * indicates significant difference between well-watered condition and drought stress under one particular concentration of DA-6.

The GP, GV, GI, and SVI greatly declined as the result of exposure to drought stress (Figures 1A–C,E). In contrast to drought-stressed seedlings, an optimal dose (2 mM) of DA-6 significantly ameliorated various germination parameters (GP, MGT, GV, GI, and SVI), however higher doses (5 and 10 mM) gradually minimized the advantageous effects of DA-6 on germination during water deficient condition (Figures 1A–E). Under water stress, seeds priming with 2 mM DA-6 exhibited 8.66% higher GP than seeds primed with distilled water (Figure 1A). Phenotypic changes showed that the DA-6 application significantly alleviated drought-induced inhibition of seeds germination (Figure 2A). Under normal condition, seed priming with various doses of DA-6 showed that 2 mM concentration significantly increased seedling FW, whereas no such difference was observed in SL and RL (Figure 2B). Under drought stress, seedling FW, SL, and RL significantly decreased in contrast to control. The DA-6 (2 mM) priming significantly mitigated the adverse effects of drought stress and resulted in higher seedling FW, RL, and SL in DA-6 primed seedlings as compared to the seedlings without DA-6 application under drought stress (Figures 2B–D).

### Effect of DA-6 on Endogenous Hormones

**FIGURE 3:**
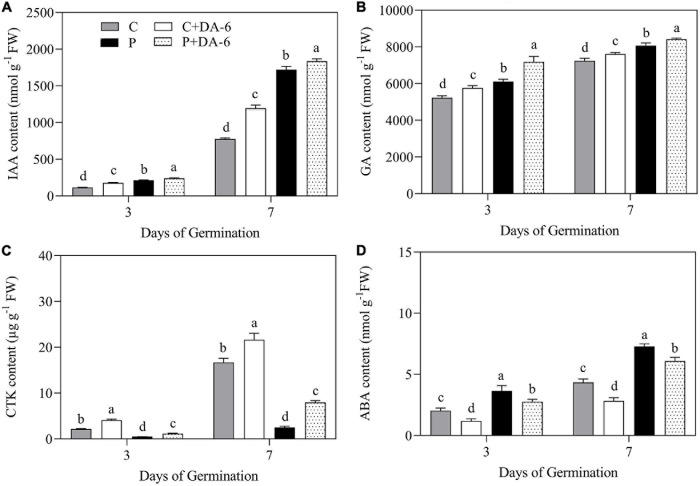
Effects of seeds priming with water or DA-6 (2 mM) on **(A)** indole-3-acetic acid (IAA) content, **(B)** gibberellin (GA) content, **(C)** cytokinin (CTK) content, and **(D)** Abscisic acid (ABA) content after 3 and 7 day of germination in white clover under well-watered condition and drought stress. Different letters above different treatments represent significant differences at a specific time point under well-watered condition or drought stress (*p* ≤ 0.05). Vertical bars show the ± SE of mean (*n* = 4).

The content of GA, CTK, IAA, and ABA were significantly influenced by seeds priming with DA-6 in seedlings during germination (3 and 7 day) under well-watered and drought conditions (Figures 3A–D). The drought stress significantly enhanced IAA, GA, and ABA content when compared to control, whereas the CTK content was significantly reduced in response to drought. Seeds priming with DA-6 (2 mM) significantly enhanced IAA, GA, and CTK content under normal and drought conditions (Figures 3A–D). The drought stress markedly increased the endogenous ABA content in seedlings at 3 and 7 days of germination, but DA-6 priming significantly inhibited ABA accumulation under normal and water stress conditions during seeds germination (Figure 3D).

### Effect of DA-6 on Starch Metabolism and Osmolyte Accumulation

**FIGURE 4:**
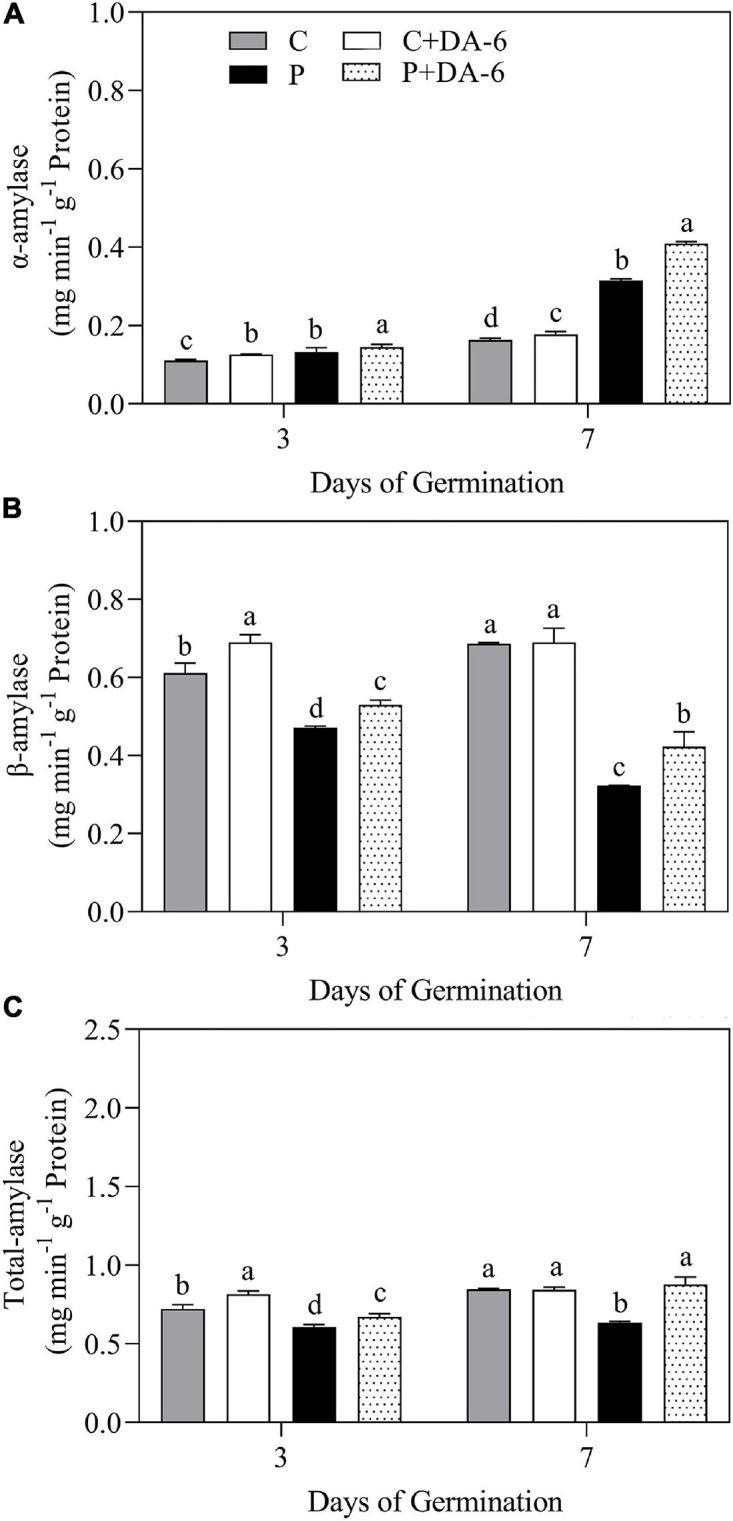
Effect of seed priming with distilled water or DA-6 (2 mM) on **(A)** α-amylase activity, **(B)** β-amylase activity, and **(C)** total amylase activity after 3 and 7 day of germination in white clover under well-watered condition and drought stress. Different letters above different treatments represent significant differences at a specific time point under well-watered condition or drought stress (*p* ≤ 0.05). Vertical bars show the ± SE of mean (*n* = 4).

**FIGURE 5:**
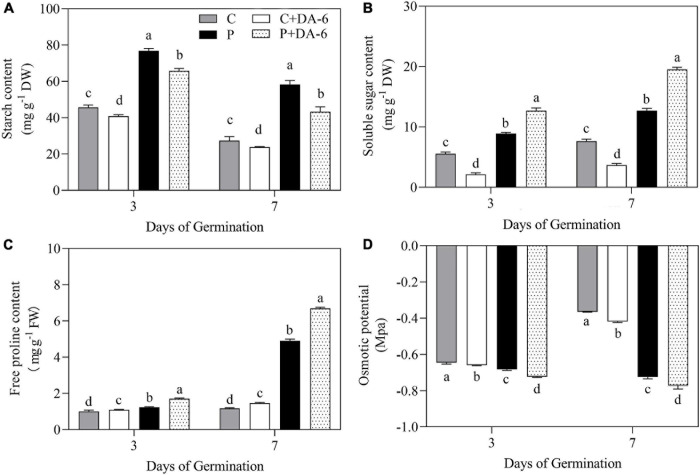
Effect of seed priming with distilled water or DA-6 (2 mM) on **(A)** starch content, **(B)** soluble sugars content, **(C)** free proline content, and **(D)** osmotic potential after 3 and 7 days of germination in white clover under well-watered condition and drought stress. Different letters above different treatments represent significant differences at a specific time point under well- watered condition or drought stress (*p* ≤ 0.05). Vertical bars show the ± SE of mean *(n =* 4).

The exogenous DA-6 application significantly improved α-amylase activity under well-watered and drought conditions (Figure 4A). Moreover, seeds priming with DA-6 significantly ameliorated drought-induced declines in β-amylase and total amylase activities at 3 and 7 days of germination under water deficient condition and also significantly enhanced β-amylase and total amylase activities at 3 d of germination under normal condition (Figures 4B,C). Drought stress significantly restrained amylolysis, resulting in higher starch content in drought-stressed seedlings compared to seedlings grown under normal condition (Figure 5A). However, DA-6 primed seedlings showed significantly lower starch content than the seedlings without DA-6 priming under normal and drought conditions (Figure 5A). Drought stress significantly increased WSC accumulation in seedlings with and without DA-6 priming (Figure 5B). The DA-6 priming substantially decreased WSC content in seedlings under normal conditions, but increased WSC content under drought stress (Figure 5B). The DA-6 priming further enhanced the WSC content by 34.90% than untreated seedlings after 7th days of drought interval (Figure 5B). The proline content and OP were substantially influenced by seeds soaking with DA-6 under well-watered conditions (Figures 5C,D). The DA-6-primed seedlings exhibited 27.43 or 26.75% higher free proline content and 5.58 or 6.12% reduced OP than non-treated seedlings at 3 or 7 days of drought stress (Figures 5C,D).

### Effect of DA-6 on Oxidative Damage and Antioxidant Enzymes Activity

**FIGURE 6:**
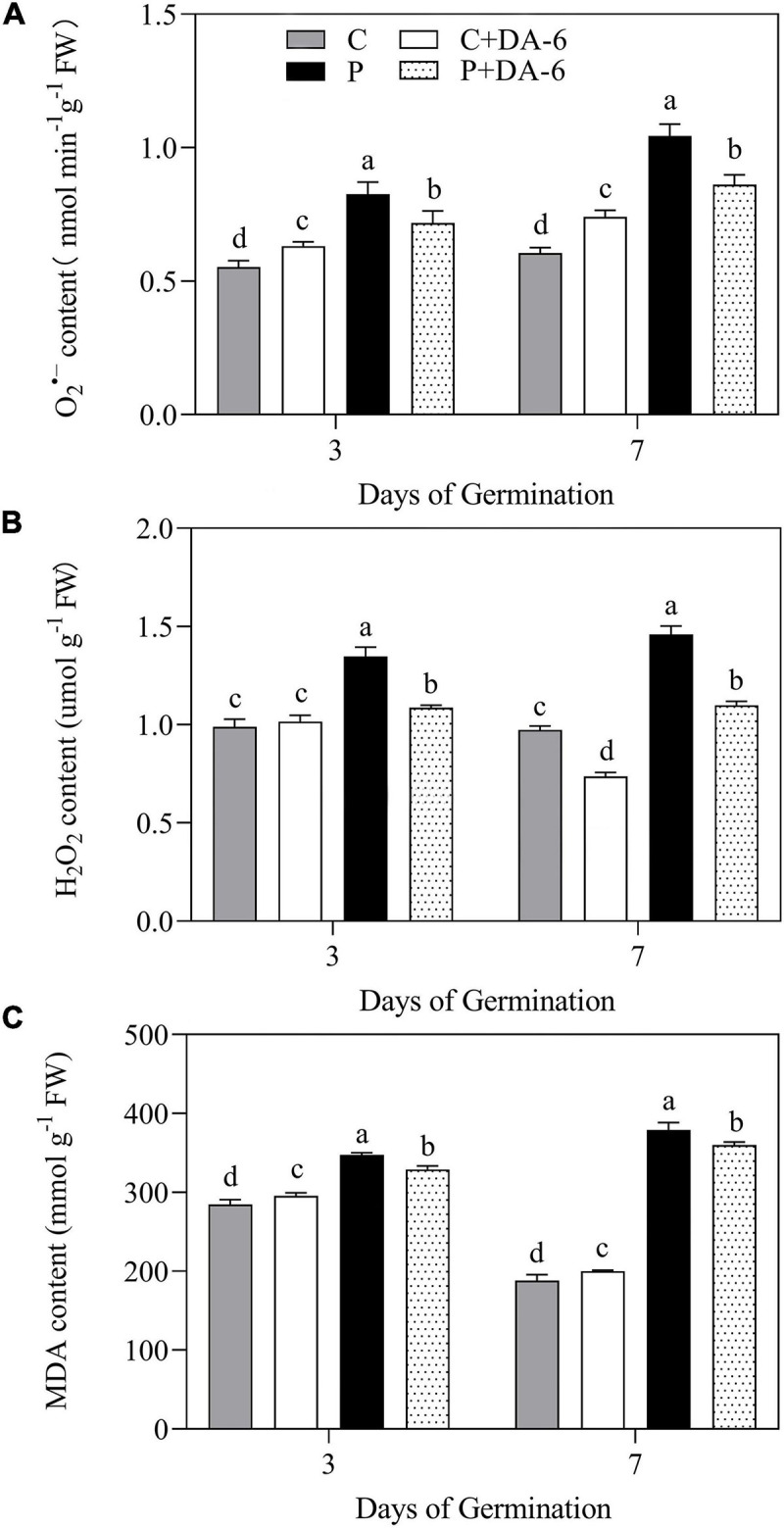
Effect of seed priming with distilled water or DA-6 (2mM) on **(A)** O_2_^–^ content, **(B)** H_2_O_2_ content, and **(C)** MDA content after 3 and 7 days of germination in white clover under well-watered condition and drought stress. Different letters above different treatments represent significant differences at a specific time point under well-watered condition or drought stress (*p* ≤ 0.05). Vertical bars show the ± SE of mean (*n* = 4).

**FIGURE 7:**
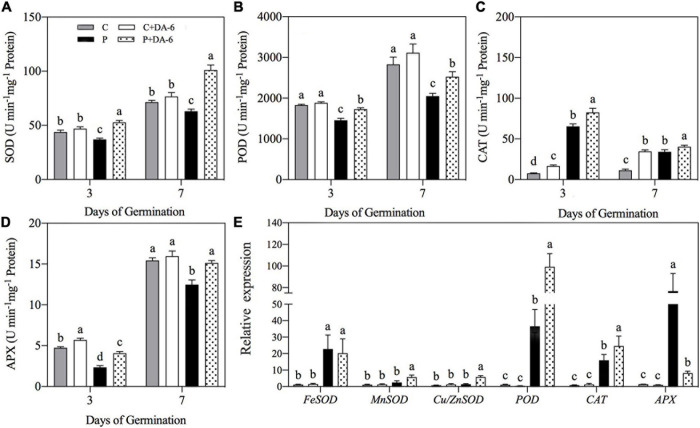
Effect of seed priming with distilled water or DA-6 (2mM) on **(A**) superoxide dismutase (SOD), **(B)** peroxidase (POD), **(C)** catalase (CAT), and **(D)** ascorbate peroxidase (APX) activities after 3 and 7 day of germination in white clover, whereas **(E)** shows expression level of antioxidant encoding genes after 3 days under well-watered condition and drought stress. Different letters above different treatments represent significant differences at a specific time point under normal condition or drought stress (*p* ≤ 0.05). Vertical bars show the ± SE of mean (*n* = 4).

Drought stress caused oxidative damage to seedlings, as indicated by elevated O_2_^∙⁣–^, H_2_O_2_ and MDA content (Figure 6). At 7th days of water stress, a 42.04, 33.28 or 49.48% increase in O_2_^–^, H_2_O_2_, or MDA content was observed in drought-stressed seedlings without DA-6 priming as compared to that in control. However, DA-6 pretreatment significantly reduced the oxidative damage through lowering the O_2_^–^, H_2_O_2_ and MDA content by 17.34, 24.66 and 5% on 7th days of water stress (Figure 6). Except CAT, the activity of all other enzymatic antioxidants (SOD, POD, APX) significantly decreased in drought-stressed seedlings without DA-6 priming in contrast to control (Figures 7A–D). DA-6-pretreated seedlings exhibited significantly higher activities of all enzymatic antioxidants than non-treated seedlings on 3rd and 7th days of water stress. In contrast to drought-stressed seedlings without DA-6 priming, the drought-stressed seedlings with the DA-6 priming demonstrated elevated enzyme activities by 37.69% for SOD (Figure 7A), 18.83% for POD (Figure 7B), 15.18% for CAT (Figure 7C), and 17.48% for APX (Figure 7D), respectively at final day of stress. During germination (3 day), the DA-6 priming did not significantly affect the enzymatic antioxidants encoding genes (*FeSOD, MnSOD, Cu/ZnSOD, POD, CAT, APX*) under well-watered conditions (Figure 7E). Except for *MnSOD* and *Cu/ZnSOD*, the drought stress significantly increased expression levels of *FeSOD, POD, CAT* and *APX* genes (Figure 7E). Seeds priming with DA-6 significantly increased transcript levels of *MnSOD, Cu/ZnSOD*, *POD*, and *CAT* in seedlings under drought stress, but the DA-6 did not have significant effect on *FeSOD* expression under normal and drought conditions (Figure 7E). In contrast to control seedlings, the drought-stressed seedlings with DA-6 priming showed significantly higher *APX*, but the expression level of this gene was lower than drought-stressed seedlings without DA-6 priming (Figure 7E). The expression level of *MnSOD, Cu/ZnSOD, POD*, or *CAT* was 2.35, 4.01, 2.71, or 1.55 times higher in drought-stressed seedlings with DA-6 priming than that in drought-stressed seedlings without DA-6 priming (Figure 7E).

### Effect of DA-6 on Dehydrins Accumulation and Expression

**FIGURE 8:**
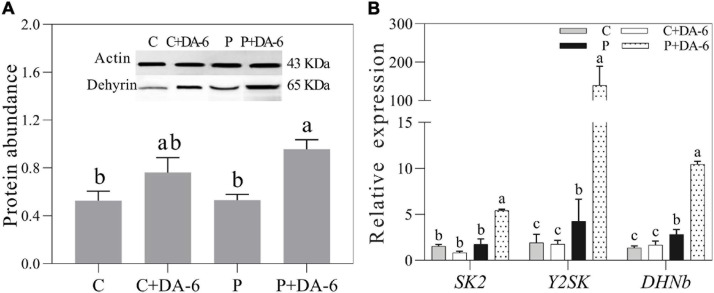
Effect of seed priming with distilled water or DA-6 (2 mM) on **(A)** Dehydrins abundance, and **(B)** *SK2* gene, *Y2SK* gene, and *DHNb* gene expression level after 3 days of germination in white clover under well-watered condition and drought stress. Different letters above different treatments represent significant difference (*p* ≤ 0.05). Vertical bars show the ± SE of mean (*n* = 4).

**FIGURE 9:**
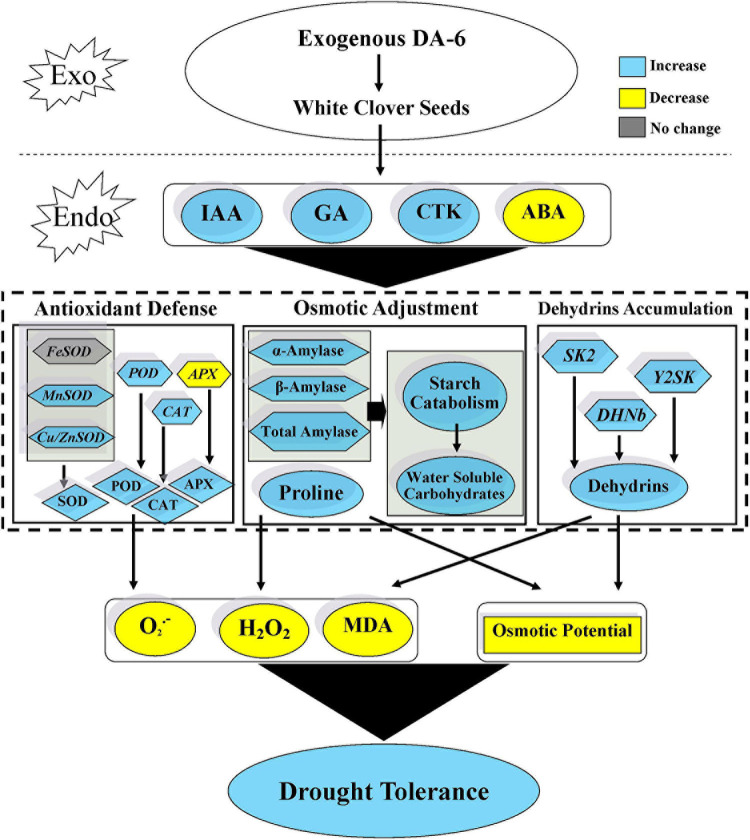
Effect of DA-6 in white clover seed germination under drought stress.

Seeds pretreatment with DA-6 induced dehydrins accumulation (65 KDa) during seeds germination under well-watered and drought conditions (Figure 8A). Under drought stress condition, seedlings primed with DA-6 showed 1.81 times higher dehydrins accumulation than the seedlings without DA-6 priming (Figure 8A). Under well-watered condition, the DA-6 showed no significant effect on expression levels of three dehydrin genes *SK2, Y2SK*, and *DHNb* (Figure 8B). The drought stress significantly up-regulated the expression levels of *Y2SK and DHNb*, while transcript level of *SK2* remained unaffected (Figure 8B). The seeds priming with DA-6 demonstrated considerably higher expression levels of all dehydrin genes (*SK2, Y2SK, and DHNb*) when compared with untreated seeds under drought stress. The expression level of *SK2, Y2SK*, or *DHNb* in DA-6 treated seedlings was 3.11, 32.87, or 3.72 times higher in contrast to untreated seedlings under drought stress (Figure 8B). Figure 9 showed a comprehensive schematic diagram representing ameliorative effect of DA-6 in white clover seeds under drought stress.

## Discussion

Drought stress significantly affects the growth and development of plants via disturbance in physiological and metabolic processes, thus restricting seeds germination and the yield of various crops (Li et al., 2014; Abid et al., 2018). Germination is among the most critical physiological processes in plants commonly regulated by phytohormones and PGRs, i.e., abscisic acid (Finkelstein and Lynch, 2000), nitric oxide (Beligni and Lamattina, 2000), melatonin (Bai et al., 2020), and DA-6 (Zhou et al., 2019). As expected, drought stress significantly decreased GP, GV, GI, SVI, RL, SL, and FW after 7 days of germination in white clover seeds (Figures 1, 2). These results were in accordance with the study of Li et al. (2014) about white clover seeds germination under water deficient conditions. Previous studies have shown that seed priming with phytohormones or PGRs is an inexpensive and useful technique to ameliorate germination and early growth and establishment of seedlings during adverse environmental conditions (Farooq et al., 2013; Biju et al., 2017). In this study, seed priming with DA-6 (2 mM) significantly increased seed GP and shortened MGT under drought stress. Moreover, DA-6 (2 mM) also exerted distinct beneficial effects by mitigating drought-induced decreases in GV, GI, SVI, FW, RL, and SL of white clover seedlings (Figures 1, 2). These results are consistent with study of Cao et al. (2018) about exogenous NaCl pretreatment during germination in white clover seeds under drought stress. The present findings indicate that seed priming with DA-6 (2 mM) could be an effective strategy to ameliorate tolerance in white clover under water deficient conditions. An earlier study found that exogenous application of DA-6 enhanced seed germination and seedling establishment by regulating fatty acids and carbohydrates metabolism in aged soyabean seeds (Zhou et al., 2019).

Various plant hormones particularly GA, IAA, CTK, and ABA perform imperative functions in seed germination (Guilfoyle et al., 2015; Liu and Hou, 2018). During seed germination, the developing embryo obtains energy from the endosperm which is composed of starch and aleurone layer (Jones and Jacobsen, 1991; Bosnes et al., 1992). The GA acts as a stimulating agent in the synthesis and production of various hydrolases including proteases, α-amylase, and β-amylase contributing to amylolysis and protein metabolism during seed germination (Appleford and Lenton, 1997; Yamaguchi, 2008). The IAA is present at radicle tip and performs vital functions in the radicle growth of emerging seedlings (Liu et al., 2007a, b). The CTK regulates a wide range of physiological processes during seed germination, especially for controlling cell division and plumule growth (Chiwocha et al., 2005; Nikolić et al., 2006; Riefler et al., 2006). Previous studies have reported that the CTK can improve seed germination by mitigating the adverse effects of heavy metals, salt, and drought stress (Khan and Ungar, 1997; Atici et al., 2005; Eisvand et al., 2010). As different from positive effects of GA, IAA, and CTK on seed germination, ABA is a primary hormone that promotes seed dormancy under drought stress (Davies and Jones, 1991). ABA exerts its negative effects on seed germination by weakening the endosperm, restricting radicle expansion, and stimulating transcriptional factors that positively regulates seed dormancy (Graeber et al., 2010; Luo et al., 2021). The current study reported that white clover seeds and seedlings demonstrated significant increase in endogenous IAA, GA, or ABA content with marked reduction in CTK content under drought stress (Figure 3). Drought induced increase in endogenous IAA, GA or ABA has been noticed in white clover (Cao et al., 2018). However, DA-6 seed priming significantly enhanced endogenous IAA, GA, and CTK content under water deficient condition (Figure 3), indicating that DA-6-regulated amelioration in germination and seedling growth related to the increases in IAA, GA, and CTK levels under water stress. Furthermore, seeds pretreatment with DA-6 significantly mitigated the drought-induced ABA accumulation, which could be one of the core reasons for alleviating drought-induced white clover seeds dormancy. Our results are consistent to a previous study where exogenous application of DA-6 improved the growth of soybean and maize seedlings by mediating photosynthesis and hormonal metabolism under normal conditions (Qi et al., 2013). These findings imply that improvement of endogenous hormonal levels (IAA, GA, CTK) by DA-6 seed priming might relate to efficient starch catabolism, osmotic adjustment and antioxidant defense system conferring enhanced tolerance in white clover under drought stress.

Starch catabolism governed by α-amylase and β-amylase is the chief physiological process in seed germination as it supplies soluble sugars (energy products) for seeds germination and growth (Appleford and Lenton, 1997; Yamaguchi, 2008). The metabolites of starch degradation such as soluble sugars are crucial for osmotic adjustment and energy supply when seeds are exposed to ionic and osmotic injury (Li et al., 2014). It has been reported that osmotic stresses such as salinity and water shortage diminished seeds germination due to the obstruction of starch catabolism (Kim et al., 2006; Li et al., 2014). During seed germination, the α-amylase is produced *de novo* in a special layer known as aleurone. In contrast, β-amylase exists in dry form in seeds as a precursor lacking catalytic activity, however, it is activated in consequence of peptide sequence cleavage at the C-terminal portion during the process of germination (Catusse et al., 2012). The present study demonstrated that water deficiency significantly inhibited starch degradation in seeds (Figure 5), but seeds primed with DA-6 considerably alleviated drought-induced decline in the process of starch catabolism through enhancing the catalytic activities of α-amylase and β-amylase under drought stress. These results inferred that DA-6-regulated stress tolerance might be associated with enhanced starch catabolism via significant improvement of hydrolases (α-amylase and β-amylase) in seeds of white clover during germination. Soluble sugars are the core constituents of cell organelles and perform important functions in osmoregulation and cellular metabolism. The current study revealed that the seeds priming with DA-6 further increased the drought-induced WSC and free proline accumulation in seedlings under water deficient conditions. Our findings are in accordance with the study of Jiang et al. (2016) about maize seedlings under saline conditions. Proline is an important osmolyte and ROS scavenger that can mitigate stress injury by lowering water potential and improving antioxidant capacity under unfavorable environmental conditions (Rathinasabapathi, 2000; Hayat et al., 2012). Our current results are parallel to the study of Zhang et al. (2016) who reported that application of DA-6 enhanced the endogenous WSC and proline content in *Cassia obtusifolia* L. resulting in improved tolerance under saline conditions. Our findings suggested that DA-6 could minimize the drought-induced damage associated with starch catabolism, WSC and proline accumulation contributing to ameliorated energy supply, OA, and osmo-protection during white clover seeds germination under water deficient condition.

Abiotic stresses disrupt the balance between production and detoxification of ROS, resulting in massive ROS accumulation conferring increased lipid peroxidation under hazardous circumstances (Li et al., 2015; Sohag et al., 2020; Luo et al., 2021). Plants contain a natural defense system comprising of enzymatic as well as non-enzymatic antioxidants to protect them from oxidative injury under unfavorable environmental conditions (Hasanuzzaman et al., 2019). SOD is the primary enzyme for the dismutation of O_2_^–^ into H_2_O_2_ and molecular oxygen (O_2_) (Luis et al., 2018). This liberated H_2_O_2_ is further detoxified by the catalytic action of POD, CAT, or ASA-GSH cycle (Fotopoulos et al., 2010; Hasanuzzaman et al., 2019). Previous studies have reported that DA-6 application improved antioxidant activities to alleviate oxidative damage in strawberry (*Fragaria* × *ananassa*) seedlings under cold stress (Fu et al., 2011), in *Cassia obtusifolia* under salt stress (Zhang et al., 2016), and in maize under low temperature stress (Zhang et al., 2020). We observed that seeds exposed to water deficient condition significantly increased O_2_^–^ and H_2_O_2_ content, however, DA-6 priming greatly alleviated the drought-induced increases in O_2_^–^ and H_2_O_2_ accumulation (Figure 6) associated with increases in the activities of antioxidant enzymes (SOD, POD, CAT, or APX) and the expression levels of genes encoding antioxidant enzymes (*FeSOD, MnSOD, Cu/ZnSOD, POD*, or *CAT*) during seed germination under water stress (Figure 7). These findings inferred that DA-6 was influential in the elimination of ROS and mitigation of membrane lipid peroxidation through improvement of antioxidant defense under drought stress. Previous findings have shown that exogenous IAA treatment enhanced endogenous IAA level, leading to the improvement of enzymatic and non-enzymatic antioxidants activity in white clover under water deficient conditions (Li et al., 2018a). Moreover, GA application during seed germination has been found to be associated with the amelioration of antioxidants defense system in rapeseed conferring tolerance under drought stress (Li et al., 2010). The study of Eisvand et al. (2010) reported that seed priming with GA and CTK enhanced enzymatic antioxidants activities in damaged seeds of tall wheatgrass under water stress. These results indicate that DA-6 induced enhancement in antioxidants defense system might be related with enhanced endogenous IAA, GA, and CTK content, thereby reducing oxidative stress injury during seeds germination under drought stress.

DHNs are special type of proteins and also recognized as LEAP (late embryogenesis abundant proteins) that are accumulated during seed germination and also produced by plants during harsh environmental circumstances, i.e., drought, cold, salinity, and heat stress (Drira et al., 2016; Marček et al., 2016). Previous studies have revealed that DHNs are involved in defense mechanisms such as the prevention of cell dehydration, OA, ROS scavenging, and membrane structure maintenance under adverse environmental conditions (Krüger et al., 2002; Hara et al., 2005; Saibi et al., 2015). Beneficial roles of DHNs accumulation and increased transcript levels of DHN genes in regulating tolerance against abiotic stresses in different plant species have been widely reported. Specific *DHN* genes (*RAB18, XERO1 and LEA14*) could prevent seed degradation under moisture deficient condition and enhance germination in *Arabidopsis thaliana* seeds under saline condition (Hundertmark et al., 2011). The current study found that the expression levels of *Y2SK* and *DHNb* were substantially up-regulated by water stress in seedlings, while the *SK2* expression did not exhibit marked difference between the control and drought-stressed seedlings without the DA-6 priming, suggesting that different types of *DHNs* behaved differently during germination under water deficient condition (Figure 8). Interestingly, seed priming with DA-6 significantly up-regulated the expression levels of all *DHN* genes (*SK2, Y2SK*, and *DHNb*) and improved the DHN (65 kDa) accumulation under drought stress (Figure 8). Similar results were found in the study of Cheng et al. (2018) who noticed that increased DHN (65 kDa) accumulation and *DHN* genes (*SK2, Y2K, Y2SK*, and *DHNb*) expression were related to γ-aminobutyric acid-induced salt tolerance during white clover seed germination. Previous studies have shown that exogenous application of ABA and CTK enhanced DHNs accumulation or transcript levels of *DHN* genes conferring tolerance to extreme environmental conditions in various plant species (Lee and Chen, 1993; Han and Kermode, 1996; Černý et al., 2011). Our findings imply that DA-6-induced drought tolerance is related to the DHNs accumulation and *SK2, Y2SK*, and *DHNb* expression. The DHNs accumulation might be regulated by alterations in endogenous GA, IAA, and CTK level induced by the DA-6, but it still deserves to be explored in our future study.

## Conclusion

In summary, the findings from the present study revealed that seed priming with DA-6 (2 mM) is a simple but efficient approach to mitigate drought-induced obstruction in seed germination. Results demonstrated that DA-6 pretreatment significantly improved the endogenous phytohormones (IAA, GA, CTK) content resulting in enhanced starch catabolism via regulation of essential hydrolases contributing toward increased OA and growth under drought stress. Moreover, DA-6 enhanced the antioxidative enzymes activities (SOD, CAT, POD, and APX) and the transcript levels of antioxidants encoding genes (*MnSOD*, *Cu/ZnSOD*, *CAT*, and *POD*) which could be associated with increased endogenous hormonal (IAA, GA, CTK) levels, thus effectively mitigating the oxidative stress injury under drought conditions. In addition, DA-6 mediated increases in dehydrins accumulation and the expression levels of dehydrin encoding genes (*SK2, Y2SK, and DHNb*) might be another vital governing mechanism conferring tolerance during germination of white clover seeds under water deficient conditions. The current study will provide a better insight about the concentration dependent role of DA-6 during seed germination under water deficient condition.

## Data Availability Statement

The original contributions presented in the study are included in the article/supplementary materials, further inquiries can be directed to the corresponding author/s.

## Author Contributions

ZL and YZ conceived, designed the research, and provided different chemical reagents and experimental material. MH, WG, WZ, and IK conducted the experiments. MH and WG evaluated the data. MH completed the manuscript writing. ZL, YP, MR, YZ, and MI reviewed and edited the manuscript. All authors contributed to the article and approved the submitted version.

## Conflict of Interest

The authors declare that the research was conducted in the absence of any commercial or financial relationships that could be construed as a potential conflict of interest.

## Publisher’s Note

All claims expressed in this article are solely those of the authors and do not necessarily represent those of their affiliated organizations, or those of the publisher, the editors and the reviewers. Any product that may be evaluated in this article, or claim that may be made by its manufacturer, is not guaranteed or endorsed by the publisher.
